# Epidemiology of Asbestosis between 2010–2014 and 2015–2019 Periods in Colombia: Descriptive Study

**DOI:** 10.5334/aogh.3963

**Published:** 2023-08-22

**Authors:** Gabriel Camero, Guillermo Villamizar, Luis M. Pombo, Manuel Saba, Arthur L. Frank, Aníbal A. Teherán, Gerhard M. Acero

**Affiliations:** 1 Cruz Roja Colombiana—Seccional Cundinamarca-Bogotá, Grupo de Investigación Emergencias, Desastres y Ayuda Humanitaria, Cruz Roja Cundinamarca y Bogotá, USA; 2 Grupo de Investigación COMPLEXUS (FUNDCLAS), Colombia; 3 Fundación Universitaria Juan N. Corpas, Grupos de Investigación COMPLEXUS, GIFVTA, Colombia; 4 Universidad de Cartagena, Facultad de Ingeniería. Grupo de Investigación de Modelación Ambiental (GIMA), Cartagena, Colombia; 5 Drexel University School of Public Health, USA; 6 Cruz Roja Colombiana—Seccional Cundinamarca-Bogotá, Grupo de Investigación Emergencias, Desastres y Ayuda Humanitaria, Cruz Roja Cundinamarca y Bogotá, Colombia

**Keywords:** Asbestosis, Colombia, Demographics, visits distribution, people attended by asbestosis

## Abstract

**Background:**

Asbestosis is a prevalent worldwide problem, but scarce data sourced from developing countries are available. We describe the sociodemographic characteristics and patterns in the occurrence of care provided for asbestosis in Colombia during the periods 2010–2014 and 2015–2019 to establish the behavior, trends, and variables associated with concentrations among people attended by asbestosis.

**Methods:**

A retrospective descriptive study was carried out with data from the Integrated Social Protection Information System (SISPRO) for two 5-year periods. People attended by asbestosis (ICD-10: J61) were identified; the frequency of patient visits, sociodemographic characteristics, case distribution patterns, and trends in both five-year periods were described, as was the crude frequency (cFr, 95% CI) of asbestosis (1,000,000 people/year) in both five-year periods (cFr ratio, 95% CI).

**Results:**

During the period 2010–2019, 765 people attended by asbestosis were identified; there were 308 people attended by asbestosis between 2010–2014 (cFr: 2.20, 1.96–2.47), and ther were 457 people attended by asbestos between 2015–2019 (cFr: 3.14, 2.92–3.50). In both periods, the estimated cFr in men was nine times the estimated cFr in women. The cFr increased in the 2015–2019 period (cFr_ratio: 1.23, 1.06–1.43). Compared with the 2010–2014 period, the cFr of asbestosis increased in women (cFr_ratio: 1.44, 1.03–2.01), in the Andean (cFr_ratio: 1.61, 1.35–1.95) and Caribbean regions (cFr_ratio: 1. 66, 1.21–2.30), in the urban area (cFr_ratio: 1.24, 1.05–1.48), and in the age groups 45–59 years (cFr_ratio: 1.34, 1.001–1.79) and ≥60 years (cFr_ratio: 1.43, 1.13–1.83).

**Discussion:**

During two five-year periods, the cFr of asbestosis was higher in men; between the first and second five-year periods, it increased significantly, especially in urbanized geographic areas and in populations aged ≥45 years. The estimates possibly reflect the effect of disease latency or the expected impact of public health policies to monitor asbestos exposure and complications.

## Introduction

Asbestosis is a public health problem with potentially catastrophic effects in terms of morbidity and quality of life, and the impact is long-term and has been documented in developed and industrially developing countries [1]. Due to the latent nature and the clinical presentation often in late stages of the disease, the characterization and identification of patterns of occurrence is essential to design preventive strategies and reduce the impact for health systems [2,3]. Persons with asbestosis have had sufficient exposure to significantly raise their risk of asbestos-related cancers.

Between 1990 and 2017, the worldwide occurrence of asbestosis increased by about 115%, the same as the incidence and prevalence, mainly in high-income countries (HIC) [4]. Heterogeneity in the estimation of incidence is high and is possibly due to the lack of epidemiological or public health studies in some countries, information biases (misclassification), underreporting, surveillance and monitoring problems in health data records, or lack of uniformity in the denominators (population or highly exposed subgroups) applied to make estimates [2,4,5,6,7].

The individual and community impact of complications associated with asbestosis was noted by the World Health Organization and the International Labor Organization, and research on exposure emerged simultaneously, revealing the latent risk in homes and industry, especially in low- and medium-income countries (LIC, MIC) that, to date, maintain unhealthy patterns of production, consumption, and final disposal of asbestos [8,9].

In Colombia, during the last decade, investigations emerged that documented the magnitude of exposure to asbestos and the effects on the health of workers, giving rise to legislative changes at the national level and prohibiting the exploitation, production, commercialization, import, and export of any asbestos variety [10,11,12,13]. However, there is still no documentation that describes the national epidemiological situation regarding asbestosis or other asbestos-related diseases in Colombia, which is the main source for making public health decisions and developing the commitments established in legislative matters.

High-income countries, not including the United States where asbestos is still a legal product, have used countrywide data to report on diseases caused by asbestos [4,8]. With the new ban in Colombia, we wish to have such data as well. Despite the chance of a misclassification bias in cases of asbestosis, the coding of the Disease Classification System (ICD-9, ICD-10) has made it possible to establish surveillance and monitoring systems to design policies aimed at controlling exposure, improving diagnostic methods, and to some extent preventing some long-term complications of asbestosis through smoking cessation, as well as flu and pneumonia vaccinations as secondary measures for patients [3,8,14]. There is really no effective treatment for asbestosis, only such secondary measures.

This research compares the epidemiological findings of asbestosis during two continuous five-year periods in Colombia (2010–2014, 2015–2019) and determines the frequency at the population level using the information contained in the database of the Integrated Social Protection Information System (SISPRO) [15].

## Methods

### Design, place of collection, and patients

A retrospective descriptive design was carried out using the ReCORD methodological standard [1]. In the database of SISPRO, records with code J61 (ICD-10, International Classification of Diseases) were identified, and information was collected on the occurrence of asbestosis in Colombia during two five-year periods, 2010–2014 and 2015–2019 [16]. The information in the SISPRO database is public and does not include data that individualizes patients with asbestosis; therefore, this research is classified as risk-free and did not require authorization by an ethics committee for research.

### Database and variables

Annual data of sociodemographic variables contained in the morbidity SISPRO platform (these modules have no restrictions on data access) were collected, including the year of occurrence, age (grouped by age cohorts), sex, health insurance (contributory, subsidiary [charity], prepaid, and others), geographic location, clinical setting, and health service provided. For each variable, people’s frequency of being attended by asbestosis, the number of annual visits (medical care provided by physicians), and the intensity of care (visits per person) was obtained [34].

### Analysis of data

The data were expressed in raw counts, proportions (percentage the number of people attended by asbestosis), and frequency (Crude rates [cFr]), estimated with the number of people attended by asbestosis (ICD-10: J61) in a specific period of time, divided by the number of visits to the whole health system in the same period of time and multiplied by 1,000,000 people (1,000,000 people year). The proportion of visits during the first period (2010–2014) was 42.8% to 60.5%, and during the second period (2015–2019), it was was 53.6% to 74% (OpenEpi, Version 3.01, released April 4 and revised April 6, 2013).

A Sankey plot (SankeyMATIC (BETA)) was used to identify case patterns between geopolitical regions and time periods. Subsequently, the risk of an asbestosis diagnosis was determined according to sociodemographic variables in the period 2015–2019, using as a reference the *cFr* estimated in the period 2010–2014. In addition, the risk of an asbestosis diagnosis in men was estimated semiannually in the years studied (OpenEpi, Version 3.01, released April 4 and revised April 6, 2013).

To identify patterns or clusters among people attended by asbestosis and the ***cFr*** of asbestosis between departments and years of study (2010–2019), a two-way cluster analysis was used. In addition, ***cFr*** of asbestosis were expressed in annual and five-year choropleth maps. Finally, to identify department groupings, an interactive cluster analysis was carried out between the ***cFr*** observed in the 2010–2014 period (*x*-axis) and the ***cFr*** observed in the 2015–2019 period (*y*-axis) (Orange Data Mining & Fruitful, Version 3.30.1).

## Results

### Frequency of asbestosis

During the period 2010–2019, 765 people attended by asbestosis were identified in the SISPRO database, with 308 in the period 2010–2014 (40.3%) and 457 in the period 2015–2019 (59.1%) (Tables 1 and 2).

**Table 1:** General characteristics among patients with asbestosis, 2010–2014.


VARIABLES	2010	2011	2012	2013	2014	PERIOD

**Frequency rate**	**2.28**	**2.76**	**1.92**	**2.04**	**3.71**	**2.20**

**95%, CI**	**1.67–3.04**	**2.12–3.53**	**1.41–2.56**	**1.51–2.70**	**3.05–4.48**	**1.96–2.47**

**People attended**	**47**	**60**	**59**	**47**	**125**	**308**

**Visits**	**121**	**123**	**106**	**89**	**223**	**662**

**Intensity** ^†^	**2.57**	**2.05**	**1.79**	**1.89**	**1.78**	**2.14**

**Life stages, years**						

<1	0 (0.0)	0 (0.0)	0 (0.0)	0 (0.0)	0 (0.0)	0 (0.0)

1–5	2 (4.6)	0 (0.0)	0 (0.0)	0 (0.0)	0 (0.0)	2 (0.66)

6–9	0 (0.0)	0 (0.0)	0 (0.0)	0 (0.0)	0 (0.0)	0 (0.0)

10–14	0 (0.0)	0 (0.0)	0 (0.0)	0 (0.0)	2 (1.9)	2 (0.66)

15–18	1 (2.3)	1 (1.7)	0 (0.0)	0 (0.0)	7 (6.8)	8 (2.7)

19–26	1 (2.3)	4 (6.8)	0 (0.0)	1 (2.2)	10 (9.7)	16 (5.4)

27–44	13 (30.2)	12 (20.2)	9 (20.4)	11 (23.9)	26 (25.2)	62 (21.0)

45–59	15 (34.9)	17 (28.8)	14 (31.8)	16 (34.8)	35 (34.0)	71 (24.1)

60+	11 (25.6)	25 (42.4)	21 (47.7)	18 (39.1)	24 (23.3)	99 (33.6)

**Sex**						

Male	39 (83.0)	52 (86.7)	44 (89.8)	39 (83.0)	80 (76.2)	254 (82.5)

Female	8 (17.0)	8 (13.3)	5 (10.2)	8 (17.0)	25 (23.8)	54 (17.5)

**Health insurance**						

Subsidiary	6 (13.9)	10 (16.9)	6 (13.6)	6 (13.0)	48 (46.6)	76 (25.8)

Contributory	29 (67.4)	43 (72.9)	37 (84.1)	40 (86.9)	51 (49.5)	200 (67.8)

Prepaid	5 (11.6)	1 (1.7)	0 (0.0)	0 (0.0)	1 (0.9)	7 (2.4)

Others	3 (7.0)	6 (10.1)	3 (6.8)	0 (0.0)	4 (3.9)	16 (5.4)

**Geographic location**						

Urban	29 (67.4)	44 (74.6)	34 (77.3)	36 (78.3)	64 (62.1)	207 (70.2)

Rural	3 (7.0)	2 (3.4)	1 (2.3)	1 (2.2)	18 (17.5)	25 (8.5)

**Clinical setting**						

Private clinics	33 (76.7)	43 (72.9)	32 (72.7)	35 (76.1)	55 (53.4)	198 (67.1)

Public hospitals	10 (23.2)	16 (27.1)	9 (20.4)	7 (15.2)	47 (45.6)	89 (30.2)

**Health service provided**						

Ambulatory	36 (83.7)	49 (83.0)	39 (88.6)	40 (86.9)	95 (92.2)	259 (87.8)

Procedure	9 (20.9)	13 (22.0)	7 (15.9)	6 (13.0)	16 (15.5)	51 (17.3)

Emergencies	1 (2.3)	6 (10.1)	2 (4.6)	3 (6.5)	2 (1.9)	14 (4.7)

In-hospital	2 (4.6)	5 (8.5)	1 (2.3)	1 (2.3)	3 (2.9)	12 (4.1)


*Note*: Frequency rate is per million people (*cFr*). **Intensity**: visits/patients assisted ratio.

**Table 2:** General characteristics among people attended by asbestosis, 2015–2019.


VARIABLES	2015	2016	2017	2018	2019	PERIOD

**Frequency rate**	**2.50**	**2.44**	**3.14**	**2.61**	**4.70**	**3.14**

**95%, CI**	**1.93–3.18**	**1.86–3.15**	**2.53–3.87**	**2.09–3.22**	**4.04–5.45**	**2.92–3.50**

**Patients assisted**	**62**	**55**	**85**	**83**	**172**	**457**

**Visits**	**187**	**148**	**160**	**189**	**237**	**921**

**Intensity** ^†^	**3.02**	**2.69**	**1.88**	**2.28**	**1.38**	**2.02**

**Life cycle, years**						

<1	0 (0.0)	0 (0.0)	0 (0.0)	0 (0.0)	0 (0.0)	0 (0.0)

1–5	0 (0.0)	0 (0.0)	0 (0.0)	0 (0.0)	0 (0.0)	0 (0.0)

6–9	0 (0.0)	0 (0.0)	0 (0.0)	0 (0.0)	0 (0.0)	0 (0.0)

10–14	0 (0.0)	0 (0.0)	0 (0.0)	0 (0.0)	0 (0.0)	0 (0.0)

15–18	1 (1.6)	0 (0.0)	0 (0.0)	0 (0.0)	3 (1.7)	4 (0.9)

19–26	4 (6.5)	1 (1.8)	2 (2.4)	5 (6.0)	10 (5.8)	22 (4.8)

27–44	19 (30.6)	11 (20.0)	12 (14.1)	17 (20.5)	39 (22.7)	98 (21.4)

45–59	14 (22.6)	18 (32.7)	33 (38.8)	21 (25.3)	40 (23.3)	126 (27.6)

60+	24 (38.7)	25 (45.5)	38 (44.7)	41 (49.4)	81 (47.1)	209 (45.7)

**Sex**						

Male	51 (82.3)	51 (92.7)	79 (92.9)	69 (83.1)	107 (62.2)	357 (78.1)

Female	11 (17.7)	4 (7.3)	6 (7.1)	14 (16.9)	65 (37.8)	100 (21.9)

**Health insurance**						

Subsidiary	1 (1.6)	11 (20.0)	9 (10.6)	19 (22.9)	95 (55.2)	135 (29.5)

Contributory	43 (69.4)	41 (74.5)	73 (85.9)	64 (77.1)	75 (43.6)	296 (64.8)

Pre-paid	1 (1.6)	1 (1.8)	2 (2.4)	0 (0.0)	1 (0.6)	5 (1.1)

Others	17 (27.4)	3 (5.5)	1 (1.2)	0 (0.0)	2 (1.2)	23 (5.0)

**Geographic place**						

Urban	46 (74.2)	36 (65.5)	65 (76.5)	65 (78.3)	149 (86.6)	361 (79.0)

Rural	5 (8.1)	5 (9.1)	5 (5.9)	9 (10.8)	13 (7.6)	37 (8.1)

Clinical settings						

Private clinics	44 (71.0)	42 (76.4)	70 (82.4)	67 (80.7)	95 (55.2)	318 (69.6)

Public hospitals	18 (29.0)	9 (16.4)	13 (15.3)	17 (20.5)	55 (32.0)	105 (23.0)

**Health service provided**						

Ambulatory	55 (88.7)	52 (94.5)	78 (91.8)	71 (85.5)	90 (52.3)	346 (75.7)

Procedure	14 (22.6)	15 (27.3)	19 (22.4)	25 (30.1)	95 (55.2)	168 (36.8)

Emergencies	1 (1.6)	3 (5.5)	2 (2.4)	1 (1.2)	0 (0.0)	7 (1.5)

In-Hospital	2 (3.2)	1 (1.8)	0 (0.0)	0 (0.0)	1 (0.6)	4 (0.9)


*Note*: Frequency is rate per million people. **Intensity:** visits/patients assisted ratio.

**Figure 1:**
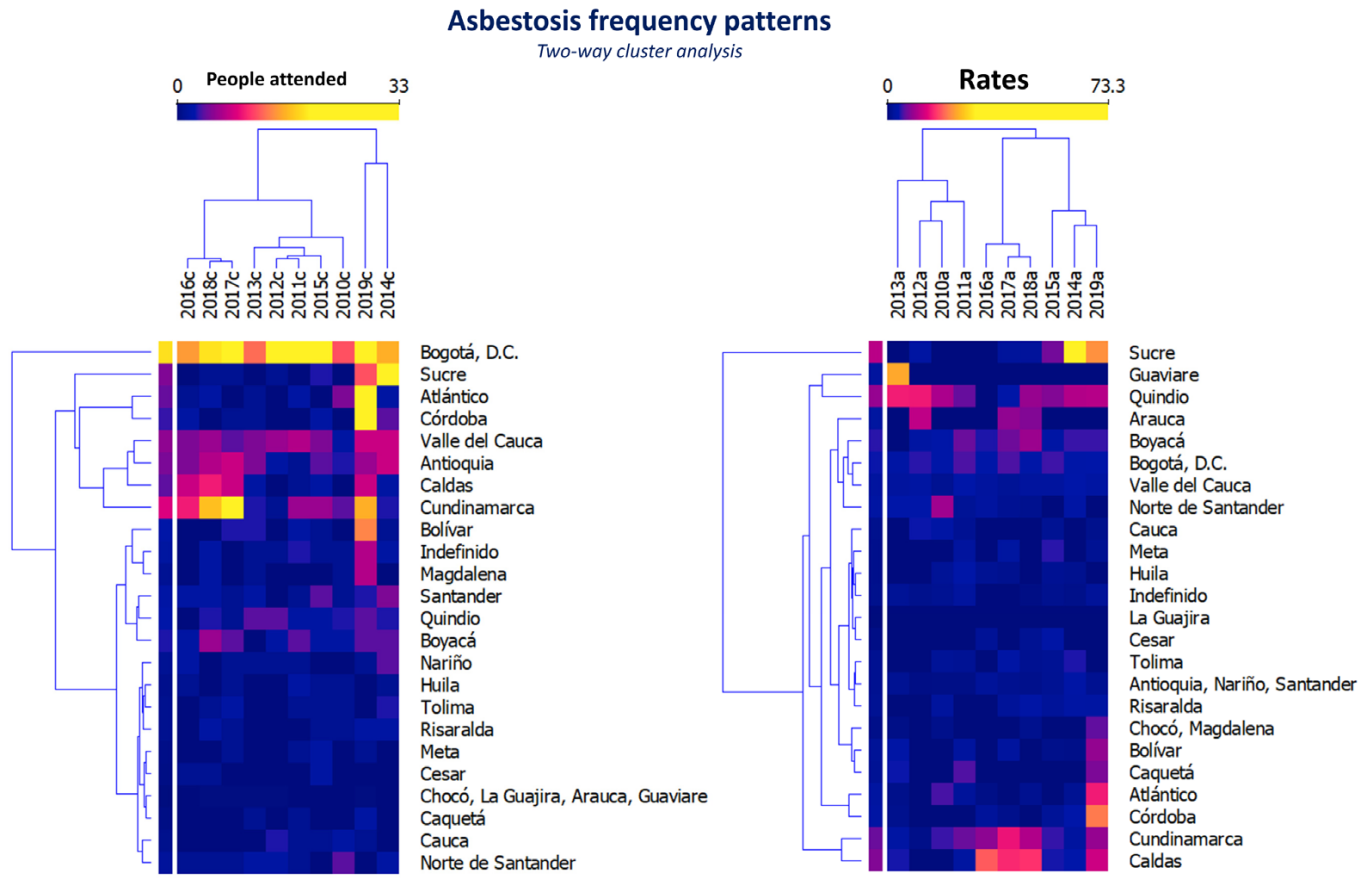
Asbestosis frequency grouped by year and department. Two dendrograms in two-way cluster analysis are presented (years of study, departments of Colombia). On the left, they are grouped by occurrence among people attended by asbestosis, and on the right, they are grouped by *cFr* rate (per million people).

As would be expected with frequency, the people attended by asbestosis increased progressively from the beginning to the end of each period and the highest occurrence was identified in the years 2014 and 2019. However, the annual occurrence was grouped into the frequencies observed in the years 2010, 2011, 2012, 2013 and 2015; Another group was made up of the frequencies of the 2016–2018 triennium, and finally, with the occurrences of the years 2014 and 2019 (Figure 1, left half). Additionally, a progressive decrease was observed in the intensity of care/visits per person per year provided in each period studied (Tables 1 and 2).

In the 2010–2014 period, about half of the people attended by asbestosis occurred in patients aged 27–59 years, about a third occurred in older people, and the remaining fraction was distributed in patients aged 15–26 years or in people attended by asbestosis where the age of the patients was unknown. However, as the population aged, half of people attended by asbestosis were now found in older people, in contrast to a third during the previous time period. In both periods, four out of every five people attended by asbestosis were men, two-thirds belonged to the contributory regime of insurance, at least one in four belonged to the subsidiary regime, and most were seen on an outpatient basis in urban areas or in private clinics (see Tables 1 and 2).

The majority of people attended by asbestosis were grouped in both study periods in the Andean region, followed by the Caribbean and Pacific regions. It was also observed that the occurrence among people attended by asbestosis in Bogotá was higher than that observed in other Colombian departments.

**Figure 2:**
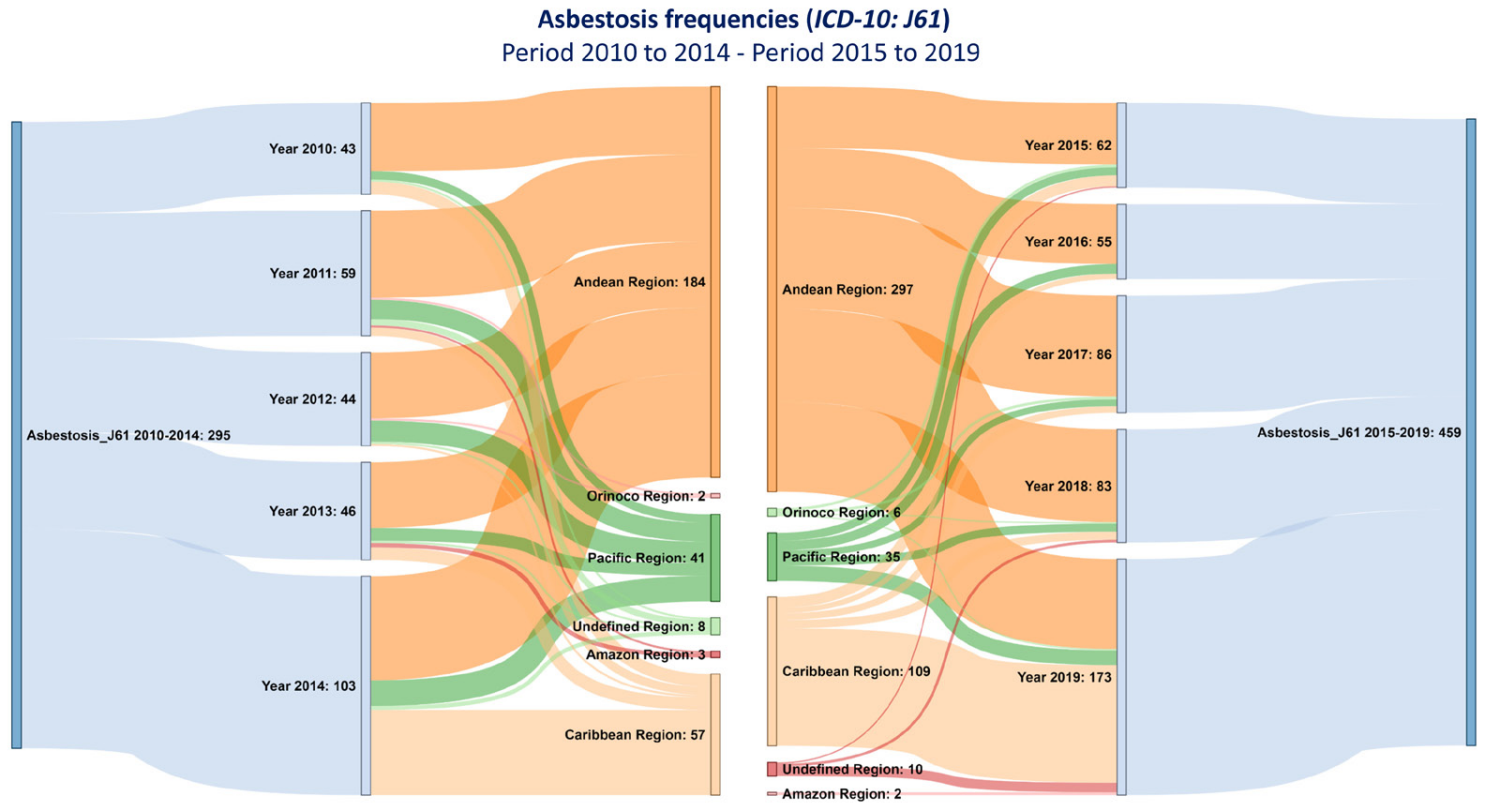
Annual and five-year occurrence of asbestosis in Colombian geopolitical regions. The occurrence among people attended by asbestosis is presented by the study periods 2010–2014 (left) and 2015–2019 (right) and by geopolitical regions.

In addition, clusters of people attended by asbestosis were identified in Sucre, Atlántico, and Córdoba; Valle del Cauca, Antioquia, Caldas, and Cundinamarca; and Bolívar, Magdalena, Santander, Quindío, and Boyacá. In the remaining departments, the occurrence in the period 2010–2019 was similar (Figure 1, left half; Figure 2).

### Asbestosis frequency estimated

**Figure 3:**
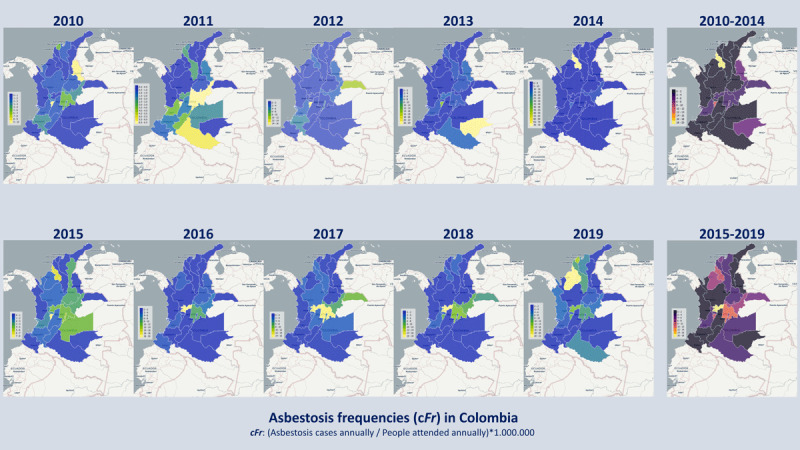
Annual and five-year dynamics of the frequency of asbestosis by departments of Colombia. Choropleth maps of the estimated frequency of asbestosis are presented in each period studied, in the upper half for the period 2010–2014 and in the lower half for the period 2015–2019.

The cFr among people attended by asbestosis per 1,000,000 people attended was higher in the period 2015–2019, and the frequency ratio showed an increase of 23% among people attended by asbestosis compared with that estimated for the period 2010–2014 (Figure 3, Figure S1). This 23% increase was greater than the five-year total population size increase.

Clusters of disease were identified in the departments of Guaviare and Quindío during the period 2010–2013. This differs from the estimated *cFr* among people attended by asbestosis, as well as in the frequencies estimated in the departments of Quindío, Arauca, and Boyacá during the 2014–2019 period. In contrast, the estimated frequencies by departments of Chocó, Magdalena, Bolívar, Atlántico, Córdoba, Cundinamarca, and Caldas were grouped in 2019, and another group of frequencies was identified in the departments of Cundinamarca and Caldas during the period 2016–2018 (Figure 1).

During the 2010–2014 period, the cFr per 1,000,000 person-years ranged between 1.92 and 3.71, and in the entire period, it was estimated between 1.96 and 2.47 (95% CI). During the 2015–2019 period, the cFr per 1,000,000 person-years ranged between 2.44 and 4.70, and in the entire period, it was estimated between 2.92 and 3.50 (95% CI).

The estimated annual cFr among people attended by asbestosis by departments during the 2010–2014 period showed a highly variable behavior, contrary to what was observed in the 2015–2019 period, where high- or medium-frequency levels in chloroplethic maps (frequency intervals) were identified in distributed departments in the Andean region, the Caribbean, and specifically the Valle del Cauca (Pacific region) (Figure 3).

### Increase in the frequency among people attended by asbestosis in the period 2015–2019

In both periods, the frequency of men treated for asbestosis was at least three times that of women. This ratio of asbestosis occurrence between men and women, with an effect directed at the former, was also evidenced when estimating the radius of cFr semiannually.

**Figure 4:**
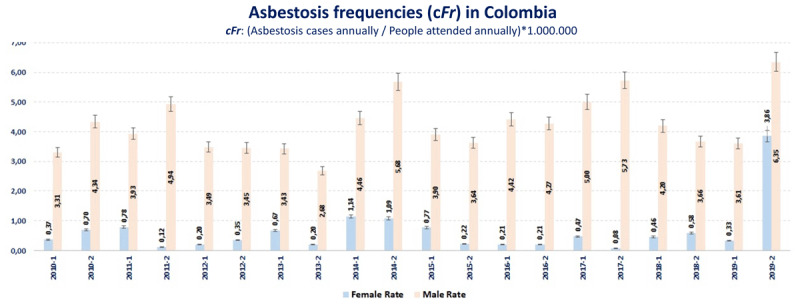
Semiannually estimated *cFr* of asbestosis by sex. The crude *cFr* among people attended by asbestosis (*y*-axis) is compared between men and women on a six-monthly basis (*x*-axis). A cyclical behavior is observed in both men and women, and in all semesters the crude *cFr* among people attended by asbestosis was higher in men.

In Figure 4 and Table S1, it is evident that the estimated cFr in men was higher than in women in all the half-year periods. However, when estimating the cFr ratio of men and women in each period studied, only in women was a significant effect evidenced, with a 44% average risk excess among people attended by asbestosis during the period 2015–2019 (Table 3).

**Table 3:** Sociodemographic characteristics and risk of asbestosis in the period 2015–2019.


PERIOD	PEOPLE ATTENDED BY ASBESTOSIS	PEOPLE ATTENDED	*cFr* (95%, CI)	*cFr* RATIO (95%, CI)

**2010–2014**				

**Sex**				

Male	254	63.192.758	4.02 (3.59–4.54)	Ref.

Female	54	92.580.322	0.58 (0.44–0.75)	Ref.

**Region**				

Andean	184	64.383.129	2.85 (2.47–3.29)	Ref.

Caribbean	57	21.852.007	2.60 (1.99–3.35)	Ref.

Pacific	41	18.096.041	2.27 (1.65–3.04)	Ref.

Orinoco	2	2.698.003	0.74 (0.12–2.44)	Ref.

Amazon	3	1.109.908	2.70 (0.68–7.36)	Ref.

**Geographic place**				

Urban	207	75.541.454	2.74 (2.38–3.13)	Ref.

Rural	25	13.154.729	1.90 (1.25–2.76)	Ref.

**Life cycle, years**				

0–9	2	23.160.474	0.09 (0.01–0.28)	Ref.

10–18	10	17.396.011	0.57 (0.29–1.02)	Ref.

19–44	78	41.798.537	1.87 (1.48–232)	Ref.

45–59	71	17.884.745	3.97 (3.12–4.98)	Ref.

60+	99	14.440.953	6.85 (5.60–8.31)	Ref.

**2015–2019**				

**Sex**				

Male	384	84.889.904	4.52 (4.09–4.99)	1.12 (0.96–1.32)

Female	101	120.576.485	0.84 (0.69–1.01)	**1.44 (1.03–2.01)**

**Region**				

Andean	297	64.234.231	4.62 (4.12–5.17)	**1.61 (1.35–1.95)**

Caribbean	109	25.188.798	4.33 (3.57–5.20)	**1.66 (1.21–2.30)**

Pacific	35	16.593.009	2.11 (1.49–2.90)	0.93 (0.59–1.46)

Orinoco	6	2.265.331	2.65 (1.07–5.50)	3.57 (0.76–25.7)

Amazon	2	965.220	2.07 (0.35–6.85)	0.77 (0.09–5.15)

**Geographic place**				

Urban	361	106.004.217	3.40 (3.06–3.77)	**1.24 (1.05–1.48)**

Rural	37	19.009.660	1.94 (1.39–2.65)	1.02 (0.61–1.72)

**Life cycle, years**				

0–9	0	27.611.772	–	–

10–18	4	20.798.824	0.19 (0.06–0.46)	0.33 (0.09–1.04)

19–44	120	52.352.834	2.29 (1.91–2.73)	1.23 (0.92–1.64)

45–59	126	23.746.715	5.31 (4.44–6.30)	**1.34 (1.001–1.79)**

60+	209	21.279.942	9.82 (8.56–11.2)	**1.43 (1.13–1.83)**


*Note*: The estimated *cFr* in the 2010–2014 period were used as a reference (Ref.) to estimate the *cFr* ratio.

Other sociodemographic characteristics in which an excess in the risk of an asbestosis diagnosis was estimated during the 2015–2019 period compared with what occurred during the 2010–2014 period were the occurrence in the Andean and Caribbean regions and in people in the ages ranges 45–59 and 60 and older.

## Discussion

This research allowed us to describe the epidemiological situation among people attended by asbestosis in Colombia during the last two five-year periods, as well as trends and patterns grouped by social and demographic characteristics.

The epidemiology of asbestosis represents what has happened in various parts of Colombia; although there is no geographically specific available data, some parts of the population have more or less active or passive exposure [2,17].

The population with passive or active exposure is essential to describe the epidemiological situation of asbestosis, because they will be the denominators used to calculate occurrences (counts, proportions) or estimate frequencies (incidence, prevalence). However, the present investigation was developed using the number of people treated in the whole health system as denominators, proposing an assumption of active and passive exposure to asbestos in periods of time prior to the period of measurement of the occurrence [7,18,19]. In addition, it is worth mentioning that the use of administrative databases as an information resource to describe the epidemiological situation of asbestosis limits the chance of expressing the results in terms of incidences, because it is unknown whether the people attended by asbestosis reported per year are new patients or old patients seen for clinical follow-up.

In Colombia between the decades of the 1940s and 1970s, the growth of industrial production and routine use of materials, equipment, automotive parts, and other products derived from asbestos began [20]. The sources of production were oriented to growth in two domains, the asbestos cement and automotive industries, responding to a demand from migratory trends, urbanization, and the extension of road networks, which was accompanied by great transformations in social and economic matters, especially in departments located in the Andean and Caribbean region [20,21,22].

Research carried out in highly urbanized cities showed high concentrations of asbestos in the air, well above the permissible limits for breathing (0.0000–0.0043 fiber/cm^3^), especially in densely built-up areas where materials derived from asbestos cement were used [23]. In urban areas and in the Colombian Andean and Caribbean regions, the highest frequency among people attended by asbestosis were estimated in the two five-year periods and analyzed. Likewise, in these geopolitical regions are the cities with the highest demographic and industrial growth—factors that stimulated internal migration to main cities and influenced the level of urbanization [24,25].

During the post–World War II period, European countries and others that made up the “British Commonwealth of Nations” were left in a catastrophic situation in terms of housing and urban planning (the decade of the 1950s). As new housing was constructed and these countries reindustrialized, considerable use was made of asbestos cement and other asbestos materials [26]. In the long term, cohort studies demonstrated the risk of mesothelioma and other diseases related to active exposure to asbestos in a young, healthy adult population that migrated from Italy to Australia during the postwar period and that was looking for work and assigned to crocidolite exploitation and extraction [26,27].

This suggests that the occurrence of asbestosis and associated complications are strongly linked to processes of social and economic transformation that occurred at least three decades previously and mainly affect the actively exposed population. As noted above, since the 1950s, Colombia experienced gradual industrial and urban growth similar to what was described in the postwar period in Europe, which could lead to the increased risk of asbestosis, as well as the development of patterns and periodic and geographic clusters such as those described in the four-year period from 2010 to 2013 and the three-year periods from 2016 to 2018, and 2014, 2015, 2019, as well as observed in Sucre, Quindío, Cundinamarca, and Caldas. Some parts of Colombia are more likely to have disease due to mining or manufacturing activities. For example, in Cundinamarca is the town of Sibaté, with a well-studied asbestos situation [28].

Among the relevant results, an increase in the frequency by people attended by asbestosis was determined from one five-year period to another, with an increased risk in women, people aged 45 or over, and in populations located in the Andean and Caribbean regions. The increase in frequency between periods may be due to the accumulation of risk between five-year periods, the implementation or improvements in diagnostic processes and epidemiological surveillance, or cohort effects such as described in the Japanese population [29,30].

Among the departments of the Andean region, Bogotá stood out with the highest occurrence of people attended by asbestosis; however, this finding may be secondary to the effects of administrative directions given the availability of highly complex health institutions where patients with asbestos-related diseases are referred because they may require multidisciplinary management and work teams highly specialized in health issues, such as lung fibrosis or mesothelioma.

Although the estimated frequency among people attended by asbestosis in men was approximately nine times that estimated in women in each five-year period, the significant risk increase in women from one period to another is striking, contrary to what was observed in men. It is possible that occupational and nonoccupational exposure to asbestos, respectively, explain the frequency among people attended by asbestosis in men and the increased risk in women [4,5,6,7,9,30,31]. Work activities generally carried out by men, such as mining and construction, have been associated with asbestosis and diseases associated with asbestos exposure. In contrast, the risk of mesothelioma and death from mesothelioma is increased among women who lived near asbestos exploitation areas (i.e., mining), and that contamination of the surroundings areas could be the mechanism; it also could occur due to “familial” or “household” exposure [27,30,31].

Distribution patterns of disease could be influenced by availability of health care and the experience of practitioners [7,18]. Another limitation that frequently occurs in research on asbestosis and associated complications is the latency period for the disease. In this case, it affects the chance of identifying events that explain the estimated frequency, but it could also influence the estimated frequency in people aged 45 years or older [2,27,28,29,32,33]. Finally, it is possible that the international guidelines, adopted in Colombia and aimed at improving the diagnosis and monitoring of asbestosis, influenced the identification of cases and estimated frequency in specific geographic locations, as observed in the department of Sucre or during the five-year period 2015–2019.

We can conclude that between the five-year periods 2010–2014 and 2015–2019, the frequency among people attended by asbestosis in Colombia increased significantly from 2.2 to 3.14 cases per million people treated in the health system. The frequency was higher in men, in people aged 45 or older, and in highly urbanized geographic areas.

We consider that the occurrence of this disease should be actively monitored. Therefore, we are working on a project that contemplates the design of a clinical guideline for nonmalignant disease due to asbestos exposure, to contribute to the process of surveillance and monitoring of this public health problem. It would be also useful in the future to monitor the effects of asbestos use by establishing appropriate oversight such as a mesothelioma registry for all of Colombia.

## Data Accessibility Statement

The research data can be found at the following link: https://doi.org/10.7910/DVN/LHFT4W [34].

## Additional File

The additional file for this article can be found as follows:

10.5334/aogh.3963.s1 Supplementary File. Figure S1 and Table S1.
