# Supplementary material for: Epidemiology of Asbestosis between 2010–2014 and 2015–2019 Periods in Colombia: Descriptive Study
Source: Ann Glob Health. 2023 Aug 22;89(1):54. doi: 10.5334/aogh.3963 (PMC10453953; doi:10.5334/aogh.3963)
Supplement: Supplementary File. — Figure S1 and Table S1. [file agh-89-1-3963-s1.pdf]

Supplementary File

Figure S1. Asbestosis frequency comparison between 2015–2019 and 2010–2014.

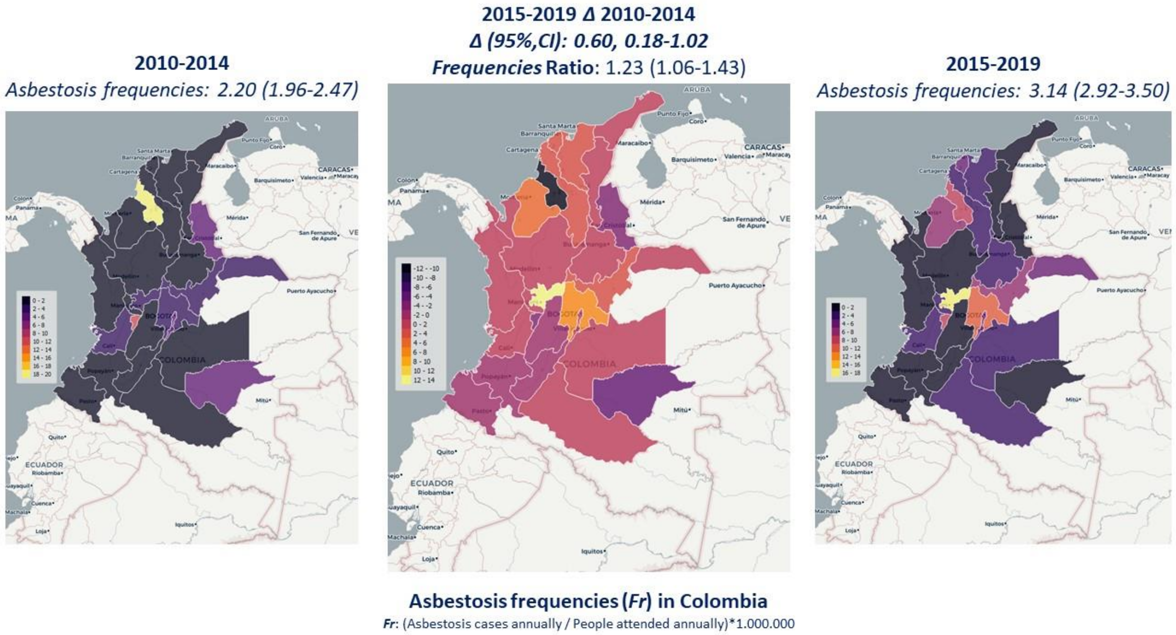

Table S1. Semiannually risk of asbestosis in men.

**Period**	**Cases**		**Number of visits**		**Prevalence ratio**
**2010-2014**	**Male**	**Female**	**Male**	**Female**	**(95%,CI)**
2010 (first semester)	18	3	5.437.190	8.098.986	8.94 (2.87-38.0)
2010 (second semester)	21	5	4.840.664	7.193.643	6.24 (2.46-18.5)
2011 (first semester)	24	7	6.107.458	8.925.609	5.01 (2.22-12.5)
2011 (second semester)	28	1	5.671.248	8.407.258	41.5 (7.86-858.0)
2012 (first semester)	24	2	6.883.511	10.132.109	17.6 (4.88-110.9)
2012 (second semester)	20	3	5.789.519	8.458.415	9.74 (3.17-41.1)
2013 (first semester)	21	6	6.119.327	8.958.679	5.12 (2.14-13.8)
2013 (second semester)	18	2	6.716.184	9.925.698	13.3 (3.56-84.8)
2014 (first semester)	32	12	7.172.774	10.512.798	3.91 (2.04-7.87)
2014 (second semester)	48	13	8.454.883	11.967.127	5.23 (2.88-10.0)
**2015-2019**					
2015 (first semester)	32	9	8.195.316	11.695.877	5.07 (2.48-11.2)
2015 (second semester)	23	2	6.325.117	9.284.261	16.8 (4.64-106.2)
2016 (first semester)	29	2	6.555.015	9.590.615	21.2 (5.97-132.0)
2016 (second semester)	28	2	6.551.209	9.438.757	20.1 (5.64-215.7)
2017 (first semester)	36	5	7.196.721	10.587.218	10.5 (4.41-30.4)
2017 (second semester)	48	1	8.372.932	12.002.639	68.8 (13.4-1403.0)
2018 (first semester)	39	6	9.288.047	13.100.327	9.17 (4.08-23.8)
2018 (second semester)	36	8	9.831.486	13.709.287	6.27 (3.01-14.4)
2019 (first semester)	40	5	11.077.748	15.352.628	11.0 (4.66-31.7)
2019 (second semester)	73	61	11.496.313	15.814.876	1.65 (1.17-2.31)

Number of visits: Number of visits to the whole health system.
